# Tortuosity of parent artery predicts in-stent stenosis after pipeline flow-diverter stenting for internal carotid artery aneurysms

**DOI:** 10.3389/fneur.2022.1034402

**Published:** 2022-10-12

**Authors:** Haibin Gao, Wei You, Dachao Wei, Jian Lv, Wei Sun, Youxiang Li

**Affiliations:** ^1^Department of Interventional Neuroradiology, Beijing Neurosurgical Institute and Beijing Tiantan Hospital, Capital Medical University, Beijing, China; ^2^Department of Neurosurgery, Beijing Boai Hospital, China Rehabilitation Research Center, Beijing, China; ^3^College of Rehabilitation, Capital Medical University, Beijing, China; ^4^Beijing Engineering Research Center, Beijing, China

**Keywords:** Intracranial Aneurysms, Pipeline embolization device (PED), vascular tortuosity, in stent stenosis, internal carotid artery (ICA)

## Abstract

**Background and purpose:**

The relationship between the tortuosity of the parent artery and treatment outcomes is not well established. We investigate the association between parent artery tortuosity and flow diverter (FD) treatment outcomes in patients with internal carotid artery aneurysms in this study.

**Methods:**

A retrospective review study was conducted to identify all patients with internal carotid artery aneurysms who were implanted with Pipeline embolization device (PED) between 2016 and 2020. The relationship between parent artery tortuosity and aneurysm complete occlusion (CO) and in-stent stenosis (ISS) was analyzed. The mathematical parameters “Curvature”, “torsion”, and “DM” extracted from the parent artery were utilized to quantify the parent artery tortuosity. A vascular narrowing of greater than 25% was categorized as ISS. Logistic regression analysis was used to identify significant independent predictors. Furthermore, we compared the performance of four machine learning algorithms and Logistic Regression model in predicting ISS.

**Results:**

This research included 62 patients who with internal carotid artery aneurysms. In 49 (79%) cases, follow-up angiography (mean follow-up duration 11.7 ±7.3 months) revealed CO of the aneurysm. ISS was detected in 22 (35.5%) cases. According to univariate analysis, parent artery tortuosity and other variables were not associated with CO (*p* > 0.1). Maximum curvature (OR = 1.084; 95% CI, 1.008–1.165; *p* = 0.03) and DM (OR = 0.01; 95% CI, 0–0.488; *p* = 0.02) exhibited strong independent associations with ISS in multivariate analysis. The SVM model is superior to the conventional Logistic Regression model and the other models in predicting ISS.

**Conclusions:**

The tortuosity of the parent artery may affect the treatment outcome of FD stenting. We found that parent artery tortuosity was associated with ISS, but not with aneurysm complete occlusion following PED stenting for internal carotid artery aneurysms in this study. Parent arteries with higher maximum curvature and lower DM were more likely to develop ISS.

## Introduction

Having gained widespread global acceptance, flow diverters (FD) have ushered in a paradigm shift in the treatment of IAs (1). The Pipeline embolization device (PED) is one of the earliest and most widely used FD and was initially approved for the treatment of internal carotid artery aneurysms (ICA) (2). A total of 83.6% of PED stents were used to treat internal carotid artery aneurysms, mostly located in the carotid siphon and carotid supraclinoid (3). Despite its short length, this segment of the artery exhibits complex morphology and marked population variation.

Tortuosity of vessels is a common angiographic finding that can be associated with vascular pathologies and may suggest systemic diseases, such as hypertension or diabetes mellitus (4–6). In terms of intracranial vasculature, tortuosity is associated with aneurysms, Moyamoya disease, and the presence of atherosclerosis (7–9). Furthermore, vascular tortuosity is associated with hemodynamic changes and vessel wall remodeling in coronary and peripheral artery studies (10, 11).

Hemodynamic changes are the main mechanism of PED stent treatment of aneurysms. ISS is a wellknown but understudied consequence after endovascular stents implantation, and was assumed to be associated with an inflammatory response due to hemodynamic status and intimal injury (12, 13). Little is known about the relationship between the degree of tortuosity of the parent artery and treatment outcomes after the PED stenting for the aneurysm. This study aimed to investigate the correlation between parent artery tortuosity and aneurysm complete occlusion (CO) and in-stent stenosis following Pipeline Flow-Diverter Stenting for Intracranial Aneurysms.

## Methods

### Study population

We retrospectively reviewed the consecutive patients with internal carotid artery aneurysms who received PED treatment at the Interventional Neuroradiology Department of our hospital from 2016 to 2020. The inclusion criteria for the study population were as follows: (1) Patients with sufficient quality of pre-stenting 3D rotational angiography (3DRA) imaging for the 3D artery model reconstruction and the analysis of tortuosity without incomplete and missing image sequence, non-standardized protocol, and severe motion artifact. (2) Patients with at least one digital subtraction angiography (DSA) follow-up for angiographic evaluations. (3) Patients who had successfully received PED implantation, while the target aneurysm without received any previous stenting and coiling before visiting our hospital. (4) Patients presenting with ideal results of parent artery reconstruction evaluated by neurosurgeons.

A total of 226 consecutive patients with internal carotid artery aneurysm who were treated with a PED stent and underwent at least one digital subtraction angiography (DSA) follow-up were retrospectively reviewed in the present study. After excluding patients without the adequate quality of pre-stenting 3D rotational angiography (3DRA) imaging (*n* = 143), 7 patients who received the previous stenting and coiling (*n* = 7) and those without idealized 3D model reconstruction of the parent artery (*n* = 14) were enrolled in this study. Patient demographics, aneurysm characteristics, procedural information, and clinical and angiographic outcomes were reviewed. This retrospective study was approved, and patient's written consent was waived off by our institutional review board.

### Endovascular procedure

The patients' treatment was started by receiving dual antiplatelet medication with aspirin 100 mg/day and clopidogrel 75 mg/day for 7 days before the implantation. Routine preoperative platelet function tests were performed, and the patients who were identified as clopidogrel non-responders received either prasugrel or ticagrelor. All PED implantations were performed under general anesthesia through a femoral approach. According to the aneurysm anatomy and based on the operator's experience, the treatment strategy was formulated to decide whether PED was to be used alone or with coiling. For patients with incomplete release of stent or incomplete stent apposition, the stent was massaged using a wire or with a balloon angioplasty. After the procedure, dual antiplatelet therapy was maintained for 6 months, and aspirin was continued indefinitely thereafter.

### Assessment of aneurysm CO and ISS

**Figure 1:**
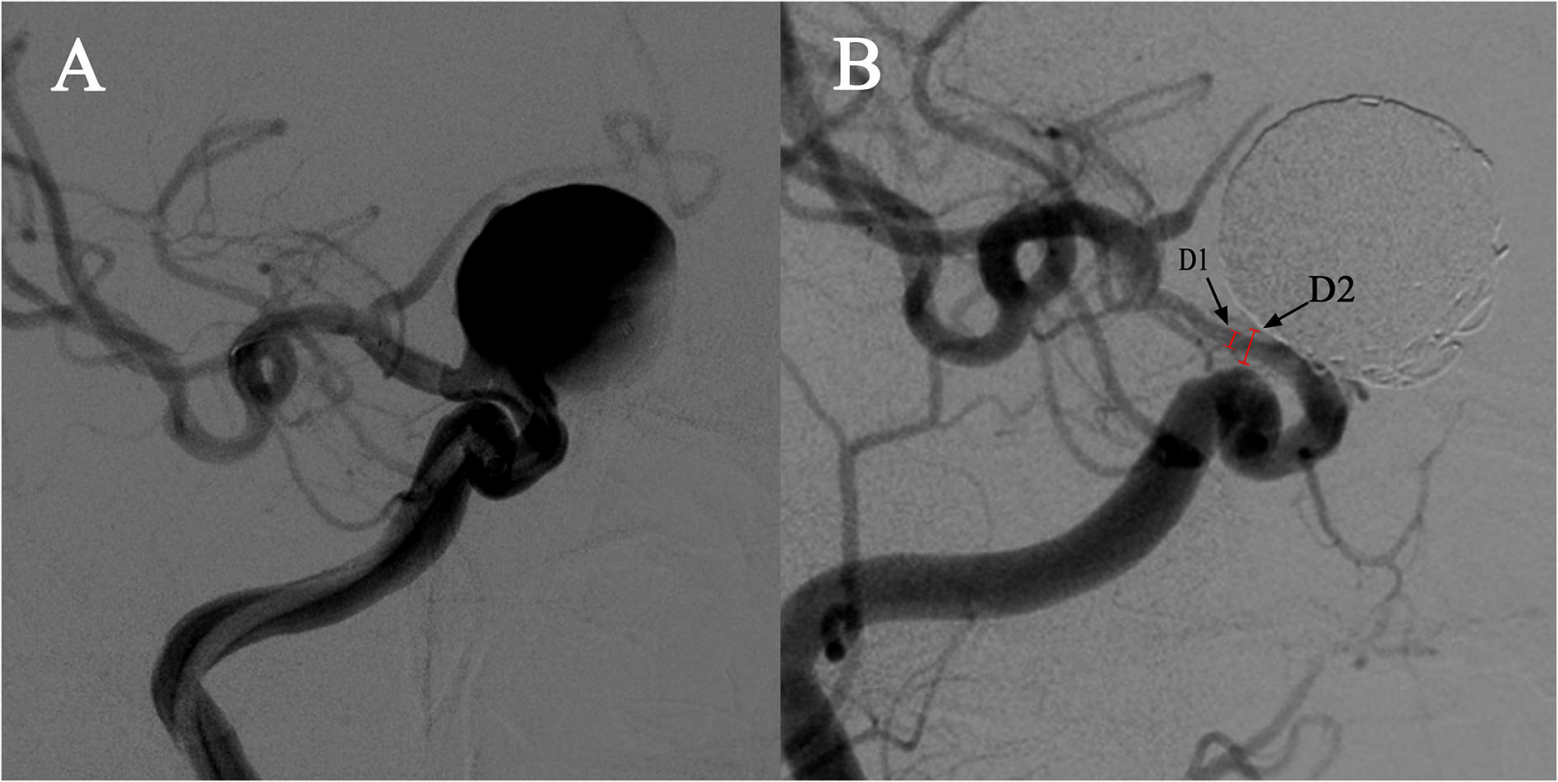
Demonstration of quantitative determination of stent stenosis. **(A)** A case of a right carotid ophthalmic aneurysm treated with a PED stent and coiling. **(B)**. The follow-up angiography showed in-stent stenosis at the distal end of the stent. D1 is the narrowest vessel diameter and D2 is the stent diameter in the same position confirmed by the mask image. The stenosis was calculated as follows: 1 – [D2/D1] × 100%.

Aneurysm occlusion was determined using follow-up digital subtraction angiography, and grading was established based on two angiographic views using the O'Kelly-Marotta grading scale (14). Follow-up DSA images were referred to assess the loss rate of the parent artery diameter, as shown in the DSA images, as the gap between the lumen vessel was filled with contrast material and the stent strut. ISS was defined as vessel narrowing of >25%. The loss rate of the parent artery diameter was calculated as follows: 1 – (vessel diameter/stent diameter) × 100% (Figure 1).

The measurement of ISS was performed by neuroradiologists with at least 3 years of experience and then reviewed by a senior neuroradiologist.

### Assessment of artery tortuosity

**Figure 2:**
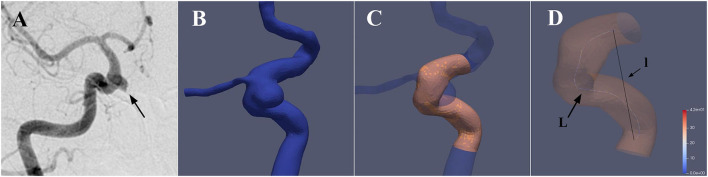
3D reconstruction of the parent artery and extraction of the vessel centerline. **(A)** A case with a right carotid ophthalmic aneurysm was treated with a PED stent. **(B)** The 3D vessel model was extracted from the raw 3D acquisition data using segmentation and surface reconstruction tools. **(C)** The aneurysm and irrelevant branch vessels were removed to get the idealized 3D reconstruction model of the parent artery, and the stent implantation segment was then identified and segmented. **(D)** Aneufuse software was used to determine the centerlines of the reconstructed 3D model. The curvature and torsion of each discrete 3D point and distance metric (DM) of the parent artery were then calculated. (l is the straight-line distance and L is the total path length)

The tortuosity analysis was based on the vessel centerline that was considered to represent the main geometric attribute of the vessel. In this study, we selected the stent implantation segment of the parent artery based on pre-stenting 3DRA imaging for artery tortuosity analyses. The mathematical parameters “Curvature”, “torsion”, and “DM” were extracted from the center line and then used to quantitatively evaluate the parent artery tortuosity (Figure 2).

#### Idealized 3D model of the parent artery

Based on the raw 3D acquisition data, the 3D model was extracted by segmentation and surface reconstruction tools based on thresholding in Mimics 19.0 (Materialize, Belgium). Smoothing operations and removal of the unrelated branching were then conducted on the 3D surface, and the domain inlets and outlets were truncated perpendicular to the centerline using the Geomagic Studio 2012 (North Carolina). The VMTK software was applied to automatically remove the aneurysm on the 3D model so as to achieve the reconstruction of the parent artery. The reconstructed 3D model was then evaluated by 2 senior interventional neuroradiologists with >5 years of experience for the successful reconstruction. Cases wherein the two neuroradiologists evaluated the results of reconstruction inconsistently were excluded from this study. The stent implantation segment of the parent artery was then identified and segmented based on the proximal and distal positions of the implanted PED stent.

#### Centerline extraction and tortuosity metrics calculation

The centerline was defined as the locus of the centers of the maximal-inscribed spheres along the vessel itself (15). The centerlines of the reconstructed 3D model were calculated automatically in the Aneufuse software as a set of discrete 3D points, which was used as an input to obtain an analytical representation through 3D freeknots regression splines (16, 17). The curvature and torsion of each discrete 3D point and the distance metric (DM) of the parent artery were calculated using a customized working program. The curvature of a curve at a point was defined geometrically as the inverse of the radius of the osculating circle at that particular point. Torsion was defined as a measure of how sharply a curve twisted out of the plane of the curvature (18). The DM quantifies the “lengthening effect” of tortuosity and was calculated as follows: DM = *l*/*L*, where *l* is the straight-line distance from the beginning point to the end point of the segment and L is the total path length of the centerline. For each parent artery, the mean curvature, maximum curvature, and range curvature were calculated. Using the same logic, the values of mean torsion, maximum torsion, and range torsion were calculated.

### Establishment of machine learning model

A total of 75 variables were included in the model establishment. Continuous variables were standardized with z-score transformation. In order to avoid class imbalance, the Boardline SMOTE algorithm was applied to the dataset. After preprocessing, the dataset was randomly split into training set (80%) and test set (20%). We used Recursive Feature Elimination (RFE) to select the best combination of ISS predictors. Then traditional Logistic Regression and four machine learning algorithms (elastic net [ENT], support vector machine [SVM], Xgboost [XGB], and random forest [RF]) were developed to predict the occurrence of ISS with the open-source machine learning library scikit-learn (version 0.24.1). Then in model training, 10-fold cross-validation and grid research were used to determine the optimal hyperparameters of the models. We compared accuracy, sensitivity, specificity, and area under the receiver operating characteristic curve (AUC-ROC) in the test set to find the best prediction model. Finally, we use the Shapley additive explanation (SHAP) algorithm (version 0.39.0) to calculate the feature importance.

### Statistical analysis

Data were presented as the frequency for categorical variables and as the mean with range for continuous variables. The Chi-square test or Fisher's exact test was applied to analyze the categorical variables, and the independent samples *t*-test was applied to analyze the continuous variables. Univariate and multivariate analyses were used to analyze the relationship between tortuosity of the parent artery and the occlusion of aneurysm and in-sent stenosis. Binary logistic regression analysis was used to identify significant independent predictors. Variables that were found to be significant at the level of 0.1 under univariate analysis or based on clinical relevance were subjected to binary logistic regression analysis. The results are presented in the form of an odds ratio (OR) and a corresponding 95% confidence interval (CI). *p* < 0.05 was considered to indicate statistical significance. A receiver operating characteristic (ROC) curve was used to analyze the performance of the logistic regression classification model. Accordingly, we performed the statistical analysis and plotted the figures using SPSS and GraphPad software.

## Results

### Patient demographics, aneurysm characteristics, procedure details, and clinical outcomes

Table 1 shows the demographics, aneurysm characteristics, and angiographic outcomes of the patients. The PED stent was used on 62 patients (mean age of 54.2 ±9.2 years; 47 females, 75.8%) with 62 targeted internal carotid artery aneurysms. All cases were saccular side-wall aneurysms, with the majority of the aneurysms found in the C6 segment (39/62, 62.9%), 11 (17.7%) in the C7, 6 (9.7%) in the C5, and 6 (9.7%) in the C4. The mean aneurysm maximum length and neck size were 9.4 ±4.7 mm and 6.2 ±2.8 mm, respectively. Ruptured aneurysms accounted for 4.8% (3/62) of cases. There were 32 (51.6%) cases treated with PED alone and 30 (48.4%) cases treated with PED plus coiling. PED Flex was used in 37 (59.7%) cases, whereas PED classic was used in the remaining cases. Multiple PED implantation was used in 8 (12.9%) procedures, while balloon angioplasty was administered in 14 (22.6%) procedures. The mean procedure duration was 121.2 ±59.5 min.

**Table 1:** Univariate analysis in association with CO of aneurysm.

**Variables**	**All (n = 62)**	**nCO (n = 13)**	**CO (n = 49)**	**p**
**Baseline demographics and clinical characteristics**				
Female, no. (%)	47 (75.8)	9 (69.2)	38 (77.6)	0.716
Age, y (mean ± SD)	54.2 ± 9.2	52.5 ± 9.1	54.7 ± 9.3	0.468
BMI	25.1 ± 3.7	26.5 ± 4.7	24.8 ± 3.3	0.13
**Co-morbidity**				
Hypertension, no. (%)	27 (43.5)	6 (46.2)	21 (42.9)	1
Diabetes, no. (%)	5 (8.1)	0 (0)	5 (10.2)	0.574
Hyperlipidemia, no. (%)	24 (38.7)	3 (23.1)	21 (42.89)	0.222
History of allergies, no. (%)	7 (11.3)	1 (7.7)	6 (12.2)	1
Smoking, no. (%)	13 (21.0)	4 (30.8)	9 (18.4)	0.444
Alcohol abuse, no. (%)	9 (14.5)	3 (23.1)	6 (12.2)	0.381
Symptomatic presentation of IA, no. (%)	36 (58.1)	10 (76.9)	26 (53.1)	0.205
Ruptured (history of SAH), no. (%)	3 (4.8)	2 (15.4)	1 (2)	0.109
**Aneurysm characteristics**				
Aneurysm neck size (mm)	6.2 ± 2.8	6 ± 2.6	6.3 ± 2.9	0.705
Maximum diameter (mm)	9.4 ± 4.7	9.3 ± 3.7	9.4 ± 4.9	0.928
Parent artery diameter (mm)	3.8 ± 0.7	3.7 ± 1	3.8 ± 0.6	0.73
Associate with parent artery stenosis, no. (%)	3 (4.8)	1 (7.7)	2 (4.1)	0.513
**Procedure characteristics**				
PED plus coiling, no. (%)	30 (48.4)	4 (30.8)	26 (53.1)	0.215
PED Flex, no. (%)	37 (59.7)	10 (76.9)	27 (55.1)	0.21
Multiple PED implantation, no. (%)	8 (12.9)	3 (23.1)	5 (10.2)	0.347
Balloon angioplasty, no. (%)	14 (22.6)	4 (30.8)	10 (20.4)	0.466
**Tortuous parameters of parent artery**				
Mean curvature	0.6 ± 0.5	0.5 ± 0.3	0.6 ± 0.5	0.564
Maximum curvature	5.3 ± 8.9	4.1 ± 4.8	5.7 ± 9.7	0.579
Range curvature	5.3 ± 8.9	4.1 ± 4.8	5.6 ± 9.7	0.579
Mean torsion	12.4 ± 4.2	11.8 ± 4.1	12.6 ± 4.3	0.563
Maximum torsion	45.4 ± 16.2	43.4 ± 15.3	45.9 ± 16.5	0.625
Range torsion	0.1 ± 0.2	43.4 ± 15.3	45.8 ± 16.6	0.635
DM	0.5 ± 0.2	0.5 ± 0.2	0.5 ± 0.2	0.823
L (total path length)	23.9 ± 9.5	25.5 ± 9.2	23.5 ± 9.7	0.507
l (straight line distance)	12.1 ± 6.2	13.5 ± 8.3	11.8 ± 5.5	0.368

In 49 (79%) cases, follow-up angiography (mean follow-up duration 11.7 ±7.3 months) revealed CO of aneurysms. ISS was detected in 22 (35.5%) lesions with a mean follow-up time of 7.8 ±4.4 months. ISS with > 50% vessel narrowing in 3 cases. There were no symptomatic cases of ISS. Treatment-related complications were observed in 3 (4.8%) cases during the periprocedural period, with one case of aneurysm rupture during the treatment procedure, one case of parenchymal hemorrhage, and one case of infarction occurring after the treatment procedure (< 24 h). There were two-thirds of cases (mRS < 2) with transient deficits and none with permanent deficits.

### Assessment of artery tortuosity

Table 1 summarizes the findings of the examination of parent artery tortuosity. The mean, maximum, and range curvatures of the parent arteries were 0.6 ±0.5, 5.3 ±8.9, and 5.3 ±8.9, respectively. Parent artery mean torsion, maximum torsion, and range torsion were 12.4 ±4.2, 45.4 ±16.2, and 45.3 ±16.2, respectively. The “L” and “l” of parent arteries were 23.9 ±9.5 and 12.1 ±6.2 mm, respectively, and the DM was 0.5 ±0.2.

### Parent artery tortuosity with CO and ISS

**Figure 3:**
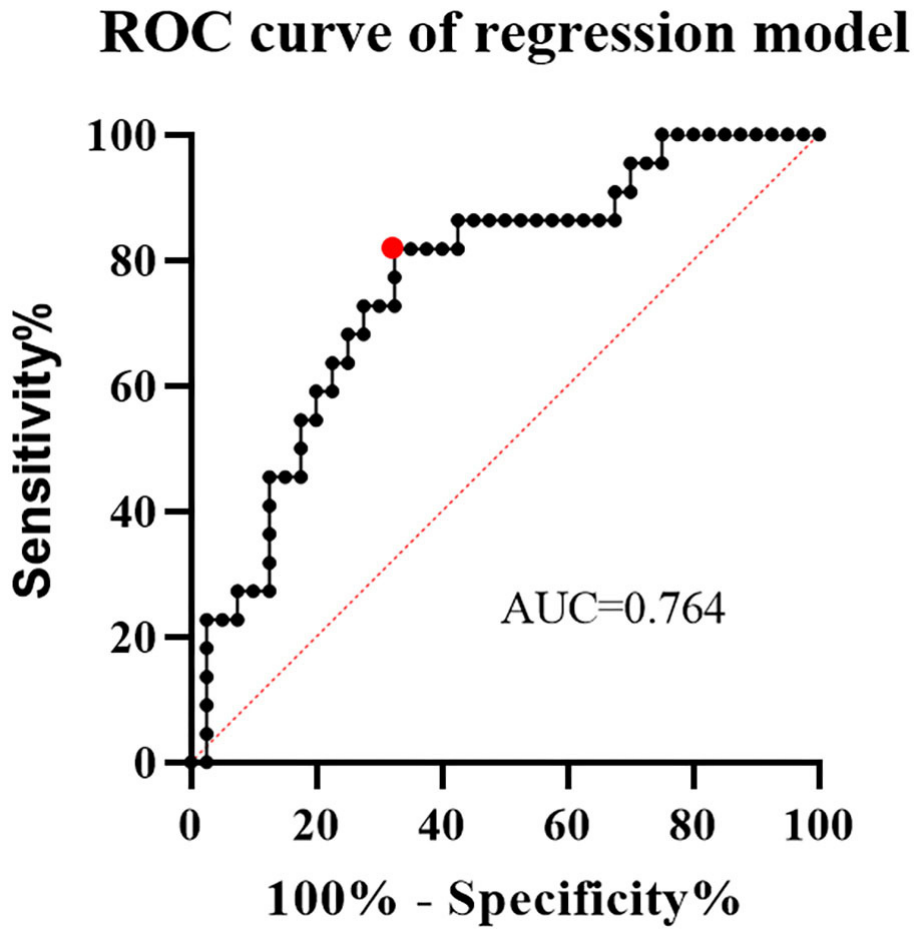
The receiver operating characteristic (ROC) curve of a logistic regression classification model. At the cutoff value, the area under the curve (AUC) was 0.764, with sensitivity and specificity of 0.82 and 0.68, respectively.

The relationship between parent artery tortuosity and the CO of aneurysm and ISS was analyzed (Tables 1, 2). Univariate analysis showed that parent artery tortuosity and other variables were not associated with CO (*p* > 0.1). The maximum curvature of the parent artery was significantly higher in individuals with ISS than in those without ISS (8.8 ±11.9 vs. 3.4 ±6; *p* = 0.021). The DM of the parent artery with ISS was significantly smaller than those without ISS (0.5 ±0.1 vs. 0.6 ±0.2; *p* = 0.021). Baseline demographics and clinical characteristics, aneurysm characteristics, and procedure characteristics did not differ significantly between patients with ISS and without ISS (p > 0.1). Significant variables in the univariate analysis that met the threshold of 10% were subjected to multivariate regression. After variables with collinearity were excluded from the collinearity test findings, mean curvature, maximum curvature, DM, and hypertension were included in the multivariate regression. In the multivariate analysis, the maximum curvature (OR = 1.084; 95% CI, 1.008–1.165; *p* = 0.03) and DM (OR = 0.01; 95% CI, 0–0.488; *p* = 0.02) exhibited strong independent associations with ISS. Specifically, parent arteries with higher maximum curvature and smaller DM were more likely to develop ISS. ROC curve was used to analyze the performance of the logistic regression classification model and the area under the curve (AUC) was 0.764 (Figure 3).

**Table 2:** Univariate and multivariate logistic analysis in association with ISS.

**Variables**	**ISS (n = 40)**	**ISS (n = 22)**	**Univariate**	**Multivariate**	
			**p**	**p**	**OR (95%CI)**
**Baseline demographics and clinical characteristics**					
Female, no. (%)	31 (77.5)	16 (72.7)	0.76		
Age, *y* (mean ± SD)	54.1 ± 8.8	54.4 ± 10.2	0.923		
BMI	25.1 ± 3.8	25.2 ± 3.6	0.906		
**Co-morbidity**					
Hypertension, no. (%)	14 (35)	13 (59.1)	0.067	0.065	—
Diabetes, no. (%)	2 (5)	3 (13.6)	0.337		
Hyperlipidemia, no. (%)	18 (45)	6 (27.3)	0.188		
History of allergies, no. (%)	4 (10)	3 (13.6)	0.989		
Smoking, no. (%)	9 (22.5)	4 (18.2)	0.756		
Alcohol abuse, no. (%)	6 (15)	3 (13.6)	1		
Symptomatic presentation of IA, no. (%)	20 (50)	16 (72.7)	0.109		
Ruptured (history of SAH), no. (%)	2 (5)	1 (4.5)	1		
**Aneurysm characteristics**					
Aneurysm neck size (mm)	6.4 ± 2.8	5.9 ± 2.8	0.567		
Maximum diameter (mm)	9.9 ± 4.7	8.5 ± 4.6	0.275		
Parent artery diameter (mm)	3.8 ± 0.6	3.7 ± 0.8	0.594		
Associate with parent artery stenosis, no. (%)	1 (2.5)	2 (9.1)	0.59		
**Procedure characteristics**					
PED plus coiling, no. (%)	22 (55)	8 (36.4)	0.192		
PED Flex, no. (%)	27 (67.5)	10 (45.5)	0.09	0.288	—
Multiple PED implantation, no. (%)	4 (10)	4 (18.2)	0.438		
Balloon angioplasty, no. (%)	9 (22.5)	5 (22.7)	1		
**Tortuous parameters of parent artery**					
Mean curvature	0.5 ± 0.3	0.8 ± 0.7	0.054	0.549	—
Maximum curvature	3.4 ± 6	8.8 ± 11.9	0.021	0.03	1.084 (1.008–1.165)
Range curvature	3.4 ± 6	8.8 ± 11.9	0.021		
Mean torsion	11.8 ± 3.7	13.5 ± 4.9	0.138		
Maximum torsion	43.8 ± 16.5	48.2 ± 15.6	0.312		
Range torsion	43.7 ± 16.5	48.2 ± 15.6	0.308		
DM	0.6 ± 0.2	0.5 ± 0.1	0.021	0.02	0.01 (0–0.488)
L (total path length)	23.7 ± 10.8	24.3 ± 6.9	0.814		
l (straight line distance)	12.9 ± 7.1	10.7 ± 3.8	0.12		

### ISS prediction using machine learning

**Figure 4:**
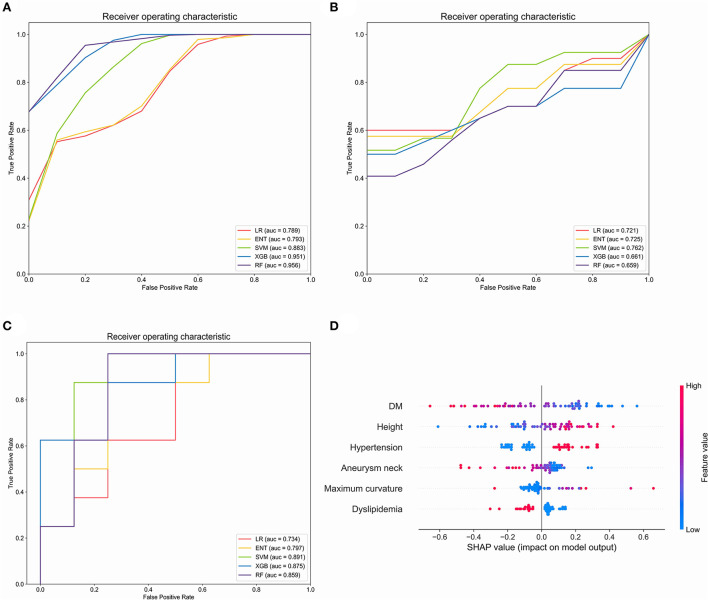
Evaluation of the machine learning models in the training set, validation set and test set. **(A)** Comparison of mean area under the receiver operating curve (AUC-ROC) in five prediction models in the training set. **(B)** Comparison of AUC-ROC in five prediction models in the validation set. **(C)** Comparison of AUC-ROC in five prediction models in the test set. **(D)** Feature importance (mean |SHAP value|) of each predictor. LR, Logistic regression; ENT Elastic net regression; Xgb, Xgboost; RF, random forest. The red or blue color of the points represents high or low feature value and the left or right of the X-axis represents positive or negative influence on the model, respectively.

In order to explore the predictive value of parent artery tortuosity on ISS, we built machine learning models. After RFE, 6 predictors (height, DM, maximum curvature, aneurysm neck, hypertension and dyslipidemia) were identified. In the training set, the RF model had the highest mean AUC-ROC (0.956; 95% confidence interval [CI], 0.951–0.961), followed by the XGB model (0.951; 95% CI, 0.943–0.959), the SVM model (0.883; 95% CI, 0.874–0.893), the ENT model (0.793; 95% CI, 0.775–0.811) and the LR model (0.789; 95% CI, 0.770–0.808) (Figure 4A). In the validation set, the SVM had the best mean AUC-ROC (0.762; 95% CI, 0.626–0.899), followed by the ENT model (0.725; 95% CI, 0.562–0.888), the LR model (0.721; 95% CI, 0.563–0.878), the XGB model (0.661; 95% CI, 0.461–0.860), the RF model (0.659; 95% CI, 0.514–0.805) (Figure 4B). In the test set, the SVM model had the best performance (0.891), with an accuracy of 87.5%, a sensitivity of 100% and a specificity of 75%, followed by the XGB model (0.875), the RF model (0.859), the ENT model (0.797) and the LR model (0.734) (Figure 4C). In SHAP analysis, DM had the greatest impact on prediction model, followed by height, hypertension, aneurysm neck, maximum curvature, and dyslipidemia (Figure 4D). Height, hypertension, and maximum curvature are the risk factors of ISS, while DM, aneurysm neck and dyslipidemia are the protective factors of ISS.

## Discussion

In the present study, we investigated the correlation between vessel tortuosity of the parent artery, in-stent stenosis, and aneurysm complete occlusion following PED Stenting for internal carotid artery aneurysm. To the best of our knowledge, this is the first study to analyze the impact of parent artery tortuosity on FD treatment results using a quantitative measurement method. “Curvature” is a parameter describing geometric curvature, “torsion” is a parameter describing the degree to which the curve torsion deviates from the osculating plane, and DM is a parameter describing the degree to which the curve deviates from the straight line. In general, more tortuous vessels have higher curvature, higher torsion, and lower DM. We discovered that vessel tortuosity was associated with ISS, but not with CO in our research. The multivariable analysis determined that higher maximum curvature and lower DM were independent predictors of in-stent stenosis. The SVM model is superior to the conventional Logistic Regression model and the other models in predicting ISS.

Previous literature has reported significant disparities in the incidence of ISS after PED implantation, which may be due to different definitions of ISS (18, 19). Some authors (20–22) considered vessel narrowing of < 25% as intimal hyperplasia or vessel narrowing of >25% as in-stent stenosis, which was consistent with our study's definition of ISS. ISS is a wellknown issue of endovascular stent implantation, although the underlying cellular mechanisms of ISS have not been welldescribed. We hypothesize that ISS formation is related to complex relationships between hemodynamics, vascular biology, and mechanical properties of the parent vessel and the stent. Previous research has revealed that abnormal vascular remodeling and neointimal hyperplasia are potential causes of ISS (23). Endothelial dysfunction caused by vascular endothelial injury leads to the proliferation of local smooth muscle cells, the formation of neointimal tissue, and eventually ISS (13). The arterial wall is invariably damaged during endovascular procedures, causing local inflammation and smooth muscle cell proliferation, leading to intimal hyperplasia and stenosis. Compared to straight arteries, stent implantation may cause greater damage to the tortuous region, resulting in fibroblastic and neointimal hyperplasia (24), leading to ISS. Additionally, the forces between the stent and the vessel wall are more complex and unstable in curved vessels than in straight vessels, which may lead to more severe vascular endothelial injury and inflammatory response.

Coronary and peripheral artery studies have shown that vascular tortuosity can affect arterial hemodynamics. Increased arterial tortuosity decreases perfusion pressure, wall shear stress, and prolonged relative residence time (25). Lowering wall shear stress can contribute to matrix metalloproteinase activation (26), leading to arterial wall remodeling (27). According to hemodynamic simulations, a curved bend disturbs the constant flow characteristic of straight vessels, resulting in complex and heterogeneous flow patterns (28, 29), which may reduce arterial endothelial function and trigger pathological degeneration of the arterial wall, which favors ISS formation (30–32).

In order to further explore the predictive value of parent artery tortuosity on ISS, we developed ISS prediction models. In contrast with traditional LR model, machine learning models can solve non-linear problem and multicollinearity, which may improve the prediction performance of the model. Therefore, we compared the performance of four popular machine learning models and LR model. The results indicate that the SVM model is superior to the conventional Logistic Regression model and the other models in terms of AUC-ROC, accuracy, sensitivity, and specificity. In SHAP analysis, DM is the most important predictor of ISS. Height, hypertension, and maximum curvature are the risk factors of ISS, while aneurysm neck and dyslipidemia are protective factors of ISS, which is consistent with the results of multivariable analysis.

The effect of FD is dependent on the induced hemodynamic changes that trigger the process of thrombosis and endothelial remodeling and ultimately seal the aneurysm. Szikora et al. (33) pointed out that the angle between the aneurysm and the parent vessel was the most important determinant of blood flow pattern in the sac. Furthermore, Xu et al. (34) showed that the hemodynamic changes of aneurysms after FD implantation were strongly dependent on the curvature of the parent artery. They found that as the curvature of the parent artery increased, the pressure, inflow velocity, and inflow volume rate also increased, while the aneurysm sac's relative residence time decreased. However, we did not find an association between parent artery tortuosity and aneurysm occlusion in this study, but this does not prove that the morphology of the parent artery does not affect the outcome of aneurysm occlusion. The arterial tortuosity parameters in this study reflect the overall tortuosity evaluation of the parent artery and may be incapable of assessing the tortuosity characteristics of specific regions, which might explain the unfavorable results.

Various methods have been proposed for the analysis of vascular tortuosity in two-dimensional (2D) and three-dimensional (3D) (10, 35). Lang and Reiter examined 89 head halves in 1984 and determined three patterns of carotid siphon morphology based on the curve angle of the artery bend (36). In 1965, Weibel and Fields proposed a classification method of internal carotid artery morphology based on the degree of vessel angulation in 2D angiography images (37). They classified the internal carotid artery vascular tortuosity patterns as Kinking, Looping, and Coiling. Many studies have examined vascular tortuosity using mathematical metrics in addition to the aforementioned assessment methods based on global vessel morphology (9, 38). To quantify vascular tortuosity, they employed parameters including the sum of angle metrics, product of angle distance, triangular index, and inflection count metrics obtained from 2D angiographic images. To evaluate vascular tortuosity, recent studies have used quantitative mathematical parameters such as tortuosity and tortuosity based on vascular reconstruction images (18, 39). In this study, 3D analysis was employed since it is more accurate and has higher consistency and accuracy than 2D analysis.

This research has certain drawbacks. First, since this is a retrospective study, it does not imply that ISS and parent artery tortuosity are causally related. To investigate the mechanism of their association, further prospective studies and laboratory evidence are needed. Second, this was a single-center retrospective study that only included patients who underwent 3D rotational angiography, which may have limited the study sample size and increased the risk of selection bias. The relatively small number of study sample may also weaken the generalization of machine learning models. Third, this study focused on the association between global vessel tortuosity and treatment outcomes, and future studies should focus on better assessing vessel tortuosity in specific regions. Despite these limitations, this is the first study to analyze the FD treatment results on parent artery tortuosity using a quantitative 3D analysis method.

## Conclusion

The tortuosity of the parent artery may affect the treatment outcome of FD stenting. We discovered that parent artery tortuosity was associated with ISS, but not with complete aneurysm occlusion after PED stenting for internal carotid artery aneurysms in this study. ISS was more common in parent arteries with higher maximum curvature and lower DM. To corroborate the current study's findings, larger cohort prospective studies and a more comprehensive assessment of vascular tortuosity are needed.

## Data availability statement

The original contributions presented in the study are included in the article/supplementary material, further inquiries can be directed to the corresponding authors.

## Author contributions

YL, WY, and HG designed the study. WY, JL, and DW contributed to data collection and data analysis. WY and HG drafted the manuscript. WS and HG performed the revision of the current literature. All authors contributed to the manuscript and approved.

## Funding

This study has received funding by the National Natural Science Foundation of China (82171289), the National Key Research and Development Program of China (2017YFB1304400).

## Conflict of interest

The authors declare that the research was conducted in the absence of any commercial or financial relationships that could be construed as a potential conflict of interest.

## Publisher's note

All claims expressed in this article are solely those of the authors and do not necessarily represent those of their affiliated organizations, or those of the publisher, the editors and the reviewers. Any product that may be evaluated in this article, or claim that may be made by its manufacturer, is not guaranteed or endorsed by the publisher.

## References

[1] Gory B Berge J Bonafe A Pierot L Spelle L Piotin M Biondi A Cognard C Mounayer C Sourour N Barbier C Desal H Herbreteau D Chabert E Brunel H Ricolfi F Anxionnat R Decullier E Huot L Turjman F Investigators D. Flow diverters for intracranial aneurysms: the diversion national prospective cohort study. Stroke. (2019) 50:3471–80. 10.1161/STROKEAHA.119.024722 31765296

[2] Ito K Kai Y Hyodo A Ishiuchi S. Long-term outcome of angioplasty or stent placement for stenosis of the cavernous or petrous portion of the internal carotid artery. Neurol Med Chir. (2011) 51:813–8. 10.2176/nmc.51.813 22198101

[3] Luo B Kang H Zhang H Li T Liu J Song D Zhao Y Guan S Maimaitili A Wang Y Feng W Wang Y Wan J Mao G Shi H Yang X. Pipeline embolization device for intracranial aneurysms in a large Chinese cohort: factors related to aneurysm occlusion. Ther Adv Neurol Disord. (2020) 13:1756286420967828. 10.1177/1756286420967828 33224273 PMC7649855

[4] Owen CG Newsom RS Rudnicka AR Barman SA Woodward EG Ellis TJ. Diabetes and the tortuosity of vessels of the bulbar conjunctiva. Ophthalmology. (2008) 115:e27–32. 10.1016/j.ophtha.2008.02.009 18423868

[5] Li Y Shen C Ji Y Feng Y Ma G Liu N. Clinical implication of coronary tortuosity in patients with coronary artery disease. PLoS ONE. (2011) 6:e24232. 10.1371/journal.pone.0024232 21904618 PMC3164184

[6] Sasongko MB Wong TY Nguyen TT Cheung CY Shaw JE Wang JJ. Retinal vascular tortuosity in persons with diabetes and diabetic retinopathy. Diabetologia. (2011) 54:2409–16. 10.1007/s00125-011-2200-y 21625945

[7] Ruan LT Duan YY Cao TS Zhuang L Huang L. Color and power doppler sonography of extracranial and intracranial arteries in Moyamoya disease. J Clin Ultrasound. (2006) 34:60–9. 10.1002/jcu.20201 16547982

[8] Kim BJ Kim SM Kang DW Kwon SU Suh DC Kim JS. Vascular tortuosity may be related to intracranial artery atherosclerosis. Int J Stroke. (2015) 10:1081–6. 10.1111/ijs.12525 26061533

[9] Klis KM Krzyzewski RM Kwinta BM Stachura K Gasowski J. Tortuosity of the internal carotid artery and its clinical significance in the development of aneurysms. J Clin Med. (2019) 8:237. 10.3390/jcm8020237 30759737 PMC6406528

[10] Ciurica S Lopez-Sublet M Loeys BL Radhouani I Natarajan N Vikkula M Maas A Adlam D Persu A. Arterial tortuosity. Hypertension. (2019) 73:951–60. 10.1161/HYPERTENSIONAHA.118.11647 30852920

[11] Hughes AD Martinez-Perez E Jabbar AS Hassan A Witt NW Mistry PD Chapman N Stanton AV Beevers G Pedrinelli R Parker KH Thom SA. Quantification of topological changes in retinal vascular architecture in essential and malignant hypertension. J Hypertens. (2006) 24:889–94. 10.1097/01.hjh.0000222759.61735.98 16612251

[12] Aguilar Perez M Bhogal P Henkes E Ganslandt O Bazner H Henkes H. In-stent stenosis after p64 flow diverter treatment. Clin Neuroradiol. (2018) 28:563–8. 10.1007/s00062-017-0591-y 28488025 PMC6245240

[13] Kipshidze N Dangas G Tsapenko M Moses J Leon MB Kutryk M Serruys P. Role of the endothelium in modulating neointimal formation: vasculoprotective approaches to attenuate restenosis after percutaneous coronary interventions. J Am Coll Cardiol. (2004) 44:733–9. 10.1016/S0735-1097(04)01083-6 15312851

[14] O'Kelly JC Krings T Fiorella D Marotta TR. A novel grading scale for the angiographic assessment of intracranial aneurysms treated using flow diverting stents. Interv Neuroradiol J Peritherapeutic Neuroradiol Surg Proced Related Neurosci. (2010) 16:133–7. 10.1177/159101991001600204 20642887 PMC3277972

[15] Archie JP Jr. Geometric dimension changes with carotid endarterectomy reconstruction. J Vasc Surg. (1997) 25:488–98. 10.1016/S0741-5214(97)70259-3 9081130

[16] Gallo D Steinman DA Morbiducci U. An insight into the mechanistic role of the common carotid artery on the hemodynamics at the carotid bifurcation. Ann Biomed Eng. (2015) 43:68–81. 10.1007/s10439-014-1119-0 25234131

[17] Gallo D Vardoulis O Monney P Piccini D Antiochos P Schwitter J Stergiopulos N Morbiducci U. Cardiovascular morphometry with high-resolution 3D magnetic resonance: first application to left ventricle diastolic dysfunction. Med Eng Phys. (2017) 47:64–71. 10.1016/j.medengphy.2017.03.011 28645847

[18] Vorobtsova N Chiastra C Stremler MA Sane DC Migliavacca F Vlachos P. Effects of vessel tortuosity on coronary hemodynamics: an idealized and patient-specific computational study. Ann Biomed Eng. (2016) 44:2228–39. 10.1007/s10439-015-1492-3 26498931

[19] Texakalidis P Bekelis K Atallah E Tjoumakaris S Rosenwasser RH Jabbour P. Flow diversion with the pipeline embolization device for patients with intracranial aneurysms and antiplatelet therapy: a systematic literature review. Clin Neurol Neurosurg. (2017) 161:78–87. 10.1016/j.clineuro.2017.08.003 28863286

[20] John S Bain MD Hui FK Hussain MS Masaryk TJ Rasmussen PA Toth G. Long-term follow-up of in-stent stenosis after pipeline flow diversion treatment of intracranial aneurysms. Neurosurgery. (2016) 78:862–7. 10.1227/NEU.0000000000001146 26600281

[21] Deutschmann HA Wehrschuetz M Augustin M Niederkorn K Klein GE. Long-term follow-up after treatment of intracranial aneurysms with the Pipeline embolization device: results from a single center. AJNR Am J Neuroradiol. (2012) 33:481–6. 10.3174/ajnr.A2790 22158922 PMC7966428

[22] Lylyk P Miranda C Ceratto R Ferrario A Scrivano E Luna HR Berez AL Tran Q Nelson PK Fiorella D. Curative endovascular reconstruction of cerebral aneurysms with the pipeline embolization device: the Buenos Aires experience. Neurosurgery. (2009) 64:632–42. 10.1227/01.NEU.0000339109.98070.65 19349825

[23] Buccheri D Piraino D Andolina G Cortese B. Understanding and managing in-stent restenosis: a review of clinical data, from pathogenesis to treatment. J Thorac Dis. (2016) 8:E1150–62. 10.21037/jtd.2016.10.93 27867580 PMC5107494

[24] Albuquerque FC Fiorella D Han P Spetzler RF McDougall CG. A reappraisal of angioplasty and stenting for the treatment of vertebral origin stenosis. Neurosurgery. (2003) 53:607–14. 10.1227/01.NEU.0000079494.87390.28 12943577

[25] Rikhtegar F Knight JA Olgac U Saur SC Poulikakos D Marshall Jr W Cattin PC Alkadhi H Kurtcuoglu V. Choosing the optimal wall shear parameter for the prediction of plaque location—a patient-specific computational study in human left coronary arteries. Atherosclerosis. (2012) 221:432–7. 10.1016/j.atherosclerosis.2012.01.018 22317967

[26] Berceli SA Jiang Z Klingman NV Pfahnl CL Abouhamze ZS Frase CD Schultz GS Ozaki CK. Differential expression and activity of matrix metalloproteinases during flow-modulated vein graft remodeling. J Vasc Surg. (2004) 39:1084–90. 10.1016/j.jvs.2003.12.031 15111865

[27] Ota R Kurihara C Tsou TL Young WL Yeghiazarians Y Chang M Mobashery S Sakamoto A Hashimoto T. Roles of matrix metalloproteinases in flow-induced outward vascular remodeling. J Cereb Blood Flow Metab. (2009) 29:1547-58. 10.1038/jcbfm.2009.77 19513084 PMC2798849

[28] Foutrakis GN Yonas H Sclabassi RJ. Saccular aneurysm formation in curved and bifurcating arteries. AJNR Am J Neuroradiol. (1999) 20:1309–17. 10.1016/j.atherosclerosis.2010.03.001 10472991 PMC7055997

[29] Zhang C Xie S Li S Pu F Deng X Fan Y Li D. Flow patterns and wall shear stress distribution in human internal carotid arteries: the geometric effect on the risk for stenoses. J Biomech. (2012) 45:83–9. 10.1016/j.jbiomech.2011.10.001 22079384

[30] Sugiyama S Niizuma K Nakayama T Shimizu H Endo H Inoue T Fujimura M Ohta M Takahashi A Tominaga T. Relative residence time prolongation in intracranial aneurysms: a possible association with atherosclerosis. Neurosurgery. (2013) 73:767–76. 10.1227/NEU.0000000000000096 23863763

[31] Riccardello GJ Jr Shastri DN Changa AR Thomas KG Roman M Prestigiacomo CJ Gandhi CD. Influence of relative residence time on side-wall aneurysm inception. Neurosurgery. (2018) 83:574–81. 10.1093/neuros/nyx433 28945849

[32] Krzyzewski RM Klis KM Kwinta BM Gackowska M Stachura K Starowicz-Filip A Thompson A Gasowski J. Analysis of anterior cerebral artery tortuosity: association with anterior communicating artery aneurysm rupture. World Neurosurg. (2019) 122:e480–6. 10.1016/j.wneu.2018.10.086 30366144

[33] Szikora I Paal G Ugron A Nasztanovics F Marosfoi M Berentei Z Kulcsar Z Lee W Bojtar I Nyary I. Impact of aneurysmal geometry on intraaneurysmal flow: a computerized flow simulation study. Neuroradiology. (2008) 50:411–21. 10.1007/s00234-007-0350-x 18180916

[34] Xu J Wu Z Yu Y Lv N Wang S Karmonik C Liu JM Huang Q. Combined effects of flow diverting strategies and parent artery curvature on aneurysmal hemodynamics: a CFD study. PLoS ONE. (2015) 10:e0138648. 10.1371/journal.pone.0138648 26398847 PMC4580450

[35] Bullitt E Gerig G Pizer SM Lin W Aylward SR. Measuring tortuosity of the intracerebral vasculature from MRA images. IEEE Trans Med Imaging. (2003) 22:1163–71. 10.1109/TMI.2003.816964 12956271 PMC2430603

[36] Lang J Reiter U. Course of the cranial nerves in the lateral wall of the cavernous sinus. Neurochirurgia. (1984) 27:93–7. 10.1055/s-2008-1053667 6483071

[37] Weibel J Fields WS. Tortuosity, coiling, and kinking of the internal carotid artery. II. relationship of morphological variation to cerebrovascular insufficiency. Neurology. (1965) 15:462–8. 10.1212/WNL.15.5.462 14288636

[38] Kim BJ Lee SH Kwun BD Kang HG Hong KS Kang DW Kim JS Kwon SU. Intracranial aneurysm is associated with high intracranial artery tortuosity. World Neurosurg. (2018) 112:e876-e880. 10.1016/j.wneu.2018.01.196 29425982

[39] Lauric A Safain MG Hippelheuser J Malek AM. High curvature of the internal carotid artery is associated with the presence of intracranial aneurysms. J Neurointerv Surgery. (2014) 6:733–9. 10.1136/neurintsurg-2013-010987 24335804

